# Metoclopramide‐Induced Pheochromocytoma Crisis: A Case Report and Literature Review

**DOI:** 10.1002/ccr3.72144

**Published:** 2026-02-26

**Authors:** Yuki Yamanishi, Kazuki Iio, Keiki Shimizu, Wataru Kasahara, Jun Hamaguchi

**Affiliations:** ^1^ Department of Emergency and Critical Care Medicine Tokyo Metropolitan Tama Medical Center Tokyo Japan; ^2^ Division of Pediatric Emergency Medicine Tokyo Metropolitan Children's Medical Center Tokyo Japan

**Keywords:** cardiac arrest, catecholamine, emergency medicine, metoclopramide, pheochromocytoma crisis

## Abstract

Pheochromocytoma is a catecholamine‐secreting neuroendocrine tumor originating in the adrenal medulla. In patients with pheochromocytoma, paroxysmal over‐secretion of catecholamines can be triggered by various medications, including the commonly used antiemetic metoclopramide. We report herein a fatal case of pheochromocytoma crisis triggered by metoclopramide and present a literature review of metoclopramide‐induced pheochromocytoma crises to elucidate their clinical characteristics.

## Key Clinical Message

Pheochromocytoma may present with vomiting, leading to inadvertent metoclopramide administration and precipitating a fatal crisis. Clinicians should maintain a high index of suspicion for pheochromocytoma when treating patients with vomiting, particularly in relatively young female patients, accompanied by suggestive symptoms such as palpitations, hypertension, or episodic catecholamine‐related manifestations.

## 1 Introduction

Pheochromocytoma is a catecholamine‐secreting neuroendocrine tumor originating in the adrenal medulla or extra‐adrenal chromaffin tissue [1]. It is extremely rare, having an estimated annual incidence of 0.6 cases per 100,000 person‐years [2]. Its diagnosis involves biochemically confirming elevated urine or serum metanephrines and identifying the tumor using imaging studies [3]. Since most pheochromocytomas are non‐metastatic, surgical excision is curative in more than 90% of the cases [4]. However, its rarity and nonspecific clinical presentation often contribute to delaying the diagnosis more than a year [5].

Patients with pheochromocytoma occasionally experience paroxysmal over‐secretion of catecholamines, which can lead to sudden‐onset hypertension or organ failure [6]. This condition, which is known as pheochromocytoma crisis, is one of the most well‐known types of endocrine emergency [6]. A review of past studies by Ando et al. found that the mortality rate associated with the most severe form of pheochromocytoma crisis was approximately 30% [7], highlighting the potentially high lethality of the condition. In addition to physical activities that exert pressure on the tumor, such as lifting heavy objects or bending forward, several medications are known to trigger pheochromocytoma crises [6]. Metoclopramide, an antiemetic commonly used in the emergency department, is among these medications [8]. Although metoclopramide is contraindicated in patients with pheochromocytoma, it may still be administered if the condition is undiagnosed at the time of presentation as nausea is a presenting symptom in 20% to 40% of cases [1]. The present report describes a fatal case of metoclopramide‐induced pheochromocytoma crisis in a patient with previously undiagnosed pheochromocytoma and includes a review of similar cases.

## 2 Case History/Examination

A 41‐year‐old female patient presented to the emergency department of a secondary referral center with vomiting. Her symptoms began with palpitations and chest tightness in the morning, followed by several episodes of vomiting around noon. She had a history of hypertension and tachycardia of several years' duration and had been taking carvedilol and amlodipine prescribed by her primary care clinic. On arrival, her vital signs were heart rate 100 beats per minute, respiratory rate 18 breaths per minute, SpO_2_ 100% on ambient air, blood pressure 133/98 mmHg, and body temperature 36.5°C. She was fully conscious at the time of her presentation. During her evaluation, intravenous metoclopramide 10 mg was administered to alleviate her nausea. Ten minutes later, her heart rate had risen to 140 beats per minute. She became agitated and cyanotic and exhibited peripheral coldness. One hour after the metoclopramide administration, her heart rate reached 170 beats per minute, and her respiratory rate increased to 40 breaths per minute. Although her systolic blood pressure remained unchanged at 139 mmHg, her diastolic blood pressure had risen to 113 mmHg. Her SpO_2_ became unmeasurable due to coldness of the extremities. She underwent tracheal intubation for respiratory failure and was transported to the study center for further management.

During transportation, the patient gradually became bradycardic, and her pulse became non‐palpable, prompting the transportation team to initiate cardiopulmonary resuscitation (CPR). Upon arrival, she had cardiopulmonary arrest, with electrocardiographic monitoring showing asystole. Spontaneous circulation was restored after 20 min of resuscitation, during which 3 mg of epinephrine in total was administered. Shockable rhythms were not observed at any point during the resuscitation. An arterial blood gas analysis on arrival revealed mixed acidosis with pH 6.99, PaCO_2_ 65.1 mmHg, HCO_3_ 15.5 mEq/L, and lactate 19.0 mmol/L. Severe hypoxemia was also noted, with PaO_2_ 74.8 mmHg on 1.0 FiO_2_. Chest X‐ray demonstrated bilateral pulmonary infiltrates, predominantly in the left lung. Echocardiography revealed apical hypokinesis with an ejection fraction of 30% indicating Takotsubo cardiomyopathy. No pericardial effusion or D‐shaped ventricular configuration was observed on echocardiography. An arterial line was placed during the initial resuscitation after cardiac arrest. Blood pressure management was thereafter guided by continuous invasive monitoring.

## 3 Differential Diagnosis, Investigations, and Treatment

**FIGURE 1:**
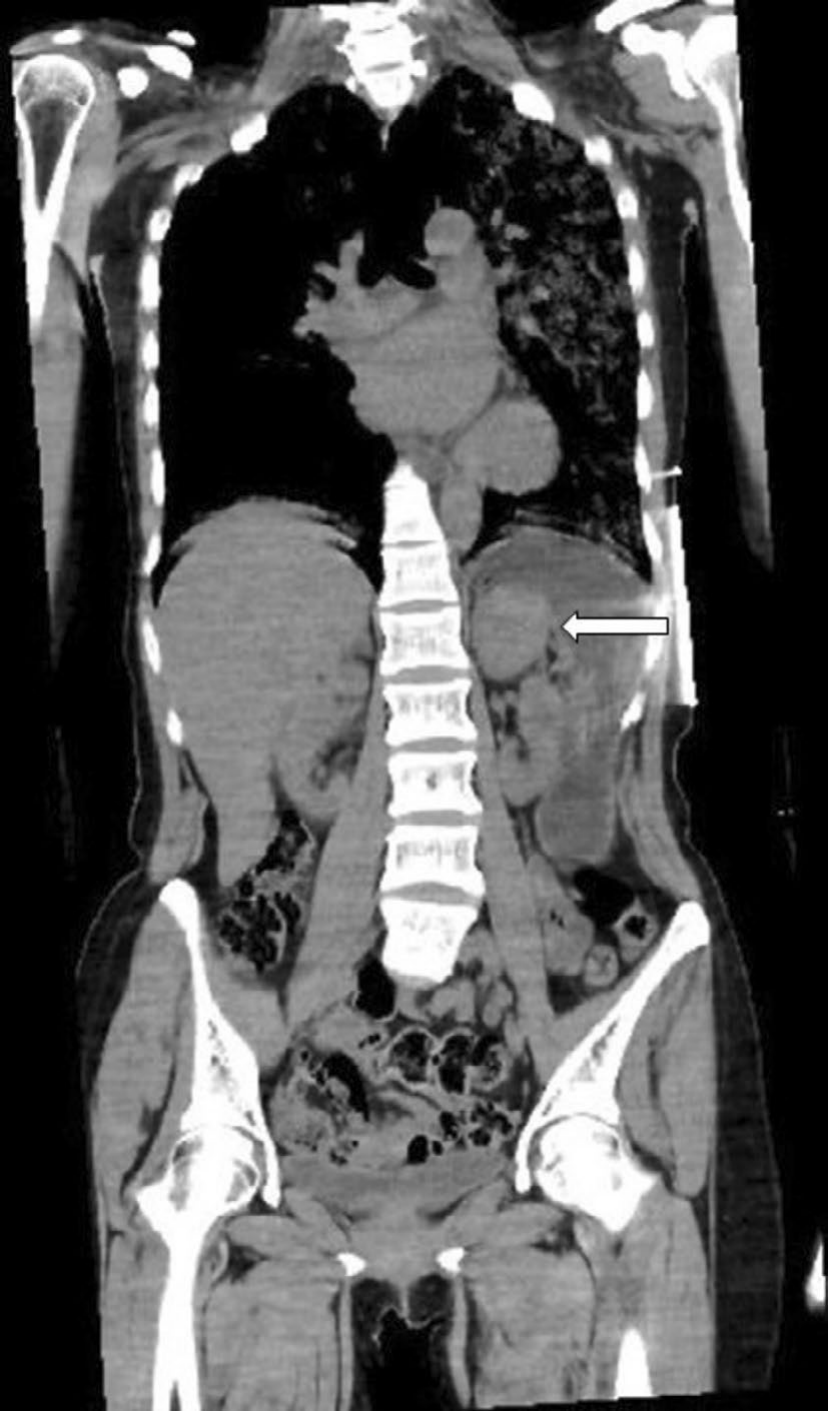
Abdominal computed tomography revealed a smooth, round tumor measuring 45 mm in diameter.

During ultrasonographic evaluation, a mass was incidentally detected on the left adrenal gland. Abdominal computed tomography (CT) revealed a smooth, round tumor measuring 45 mm in diameter (Figure 1). Given that the tumor was found in the context of rapid respiratory and cardiac deterioration following the administration of metoclopramide, a pheochromocytoma crisis was strongly suspected.

Intensive care, including target temperature management for post‐cardiac arrest syndrome, was begun. Acute normalization of blood pressure was not necessary in our case as blood pressure returned to the normal range after resuscitation. We initiated a continuous infusion of phentolamine mesylate and titrated the dose as needed to control hypertension. A landiolol hydrochloride infusion was added subsequently to control tachycardia. The serum‐free metanephrine and normetanephrine tests conducted on the day of her arrival returned 1 week later, showing a marked elevation in the metanephrine and normetanephrine value to 52.3 nmol/L and 106.5 nmol/L, respectively.

## 4 Outcome and Follow‐Up(Conclusion and Results)

Head CT performed 72 h after the start of targeted temperature management found evidence of severe hypoxic encephalopathy. Given her poor neurological prognosis, the tumor was not resected. After discussing with her family, she was extubated, and continuous morphine administration was begun to alleviate her respiratory discomfort. She passed away on day 18 of illness. In accordance with the family's wishes, no autopsy was performed.

## 5 Discussion

Pheochromocytoma was unable to be diagnosed definitively in the present case owing to the lack of pathological information. However, the patient's serum free metanephrine and normetanephrine levels were 50 times higher than the upper reference value [9], strongly suggesting that the adrenal tumor detected during the evaluation was a catecholamine‐secreting pheochromocytoma. Since no medications had been administered until metoclopramide was given, we suspect that metoclopramide exacerbated the pheochromocytoma crisis in this case and led to the development of Takotsubo cardiomyopathy and pulmonary edema, which ultimately caused the hypoxemia, leading to cardiac arrest.

Excessive catecholamine release in pheochromocytoma can result in a wide spectrum of cardiovascular complications, including myocardial ischemia, acute heart failure, arrhythmias, and stress‐induced cardiomyopathy [10]. Proposed mechanisms of catecholamine‐mediated myocardial injury include coronary vasospasm, increased myocardial oxygen demand, direct myocardial toxicity, and microvascular dysfunction [10]. Shahriar et al. reported a rare case of pheochromocytoma‐induced myocardial ischemia and emphasized that excessive catecholamine release can cause ischemic myocardial injury even in the absence of significant coronary artery disease [10]. In the present case, echocardiography revealed apical hypokinesis with reduced left ventricular ejection fraction, which was consistent with Takotsubo cardiomyopathy. This finding supports the concept that acute catecholamine surges during pheochromocytoma crisis can induce reversible myocardial stunning and ischemic myocardial injury.

Metoclopramide, a D2 receptor antagonist frequently used as an antiemetic in emergency departments [11], was incidentally identified as an inducer of pheochromocytoma crisis in 1976 [12]. Since then, several case studies of metoclopramide‐induced pheochromocytoma crises have been published. To clarify the clinical characteristics of pheochromocytoma crises induced by metoclopramide, we conducted a review of past studies using PubMed with the search terms, “pheochromocytoma” and “metoclopramide.” The review of 11 documents describing the clinical course of 13 patients [8, 12, 13, 14, 15, 16, 17, 18, 19, 20, 21] included case reports and case series published between 1976 and 2024 in English. Experimental studies, such as those using metoclopramide as a diagnostic agent for pheochromocytoma [22], were excluded.

Table 1 summarizes the details of the reports. Most of the patients were female, and the most common age range was 30 to 40 years. Two patients were pregnant when they experienced a pheochromocytoma crisis, making it difficult to distinguish the condition from pre‐eclampsia. In most cases, metoclopramide had been inadvertently administered because pheochromocytoma had not been diagnosed at the time of the presentation. Notably, more than half the patients experienced preceding symptoms lasting weeks or even years. Although most patients exhibited at least one symptom from the classic triad consisting of headache, diaphoresis, and palpitations, a near‐fatal case reported by Xie et al. showed no typical symptoms of pheochromocytoma throughout the clinical course [8]. Three cases, including ours, resulted in death from a pheochromocytoma crisis.

**TABLE 1:** Clinical characteristics of the patients included in the literature review.

	Age, sex	Pregnancy status	Country	Known PPGL	Symptoms at presentation	Symptoms before presentation	Response to metoclopramide	Treatment, prognosis
Plouin et al. (1976)	—	—	France	Yes	Nausea (during angiography)	—	Systolic BP rose to 340 mmHg.	Tumor resection
Freestone et al. (1996)	34, F	No	UK	No	Episodic dizziness, sweating, recurrent headaches, and nausea	Poor blood pressure control in the last few weeks	Became unwell, clammy, and pale. BP rose to 280/160 mmHg	Tumor resection
Takai et al. (1997)	37, F	Yes	Japan	No	Nausea, vomiting, headache, and palpitation	Similar episodes in the last 6 years	Rise in blood pressure and pulmonary edema development	Tumor resection
Leow et al. (2005)	30, M	—	Singapore	No	Vomiting and abdominal pain	Episodic headache and abdominal pain for the past several months	Hypertensive spell followed by profound shock refractory to vasopressor	Tumor resection
Leow et al. (2005)	27, M	—	Singapore	No	Vomiting and abdominal pain	Presentation to the ER with similar symptoms a month earlier	BP rose to 190/100 mmHg. Another dose resulted in severe headache, diaphoresis, and palpitation.	Tumor resection
Leow et al. (2005)	61, F	No	Singapore	Yes	Vomiting and anorexia	Paroxysmal spells of headaches, palpitations, and labile BP	BP rose to 270/130 mmHg. Headache, diaphoresis, and palpitation developed.	Declined surgery
Guillemot et al. (2009)	46, F	No	France	No (Strongly suspected)	Nausea	History of labile hypertension and paroxysmal headaches, palpitations, and cold sweating	Rise in blood pressure, profound cardiac hypokinesia and left ventricular thrombus formation	Tumor resection
Frankton et al. (2009)	47, M	—	UK	No	Hypoglycemia, vomiting, abdominal pain, and profuse sweating	Vomiting for 4 days before presentation with no earlier manifestations	Extremely hypertension and hypoglycemia recurrence	Deceased
Sheinberg et al. (2012)	45, F	No	US	No	Nausea, vomiting, headache, and palpitation	Three‐month history of migraine headache	Acute hypertension, diaphoresis, and severe Takotsubo myopathy requiring IABP and VA‐ECMO developed.	Deceased (possible complication by IABP)
Leonard et al. (2018)	36, F	No	US	No	Nausea, vomiting, and headache	—	BP rose to 223/102 mmHg. Severe cardiogenic shock and ARDS requiring VV‐ECMO developed.	Tumor resection
Lazari et al. (2021)	63, F	—	UK	No	Gradual onset lower back discomfort, intermittent headache, nausea, sweating, and dyspnea	—	Post‐ coronary angiography nausea and vomiting were followed by respiratory failure. BP reached 350/140 mmHg after metoclopramide administration.	Tumor resection
Negro et al. (2021)	33, F	Yes	Italy	No	Headache and increased blood pressure (220/120 mmHg)	Blood pressure control became poor from 10 weeks before presentation.	Oral metoclopramide prescribed 1 week before presentation led to symptom onset.	Tumor resection
Xie et al. (2023)	46, F	No	China	No	Nausea and vomiting after intrauterine device removal	Previously in good health	Diastolic hypertension and hypoxemia were followed by Takotsubo cardiomyopathy and respiratory failure requiring VA‐ECMO.	Tumor resection
Present case (2025)	41, F	No	Japan	No	Vomiting, chest tightness, and palpitation	Unknown	Increased respiratory rate, heart rate; cardiac arrest after tracheal intubation	Deceased

Abbreviations: BP, blood pressure, ER, emergency room; IABP, intra‐aortic balloon pumping; PPGL, pheochromocytoma and paraganglioma; VA‐ECMO, veno‐arterial extracorporeal membranous oxygenation; VV‐ECMO, veno‐venous extracorporeal membranous oxygenation.

Given the potentially serious consequences of a pheochromocytoma crisis, pheochromocytoma should ideally be suspected in all patients presenting to the emergency department with vomiting. However, the imbalance between the large number of ER visits for vomiting or nausea and the extremely rare occurrence of pheochromocytomas makes this task quite challenging. The presence of preceding, paroxysmal symptoms, as observed in our review, may help raise suspicion of pheochromocytoma in patients with vomiting. Several studies have suggested an association between physical activity and nausea in patients with pheochromocytoma [23, 24], which may serve as another clue. Nevertheless, these indicators are inadequate to rule out pheochromocytoma before administering metoclopramide. Notably, our literature review suggests that metoclopramide‐induced pheochromocytoma crises tend to occur more frequently in relatively young female patients, a population in whom pheochromocytoma may not be readily suspected in emergency settings. Therefore, physicians should remain mindful of pheochromocytoma whenever they consider prescribing metoclopramide to a patient with vomiting. For the prompt recognition of a pheochromocytoma crisis, physicians should remain mindful of pheochromocytoma whenever they consider prescribing metoclopramide to a patient with vomiting.

## Author Contributions

**Yuki Yamanishi:** writing – original draft. **Kazuki Iio:** writing – review and editing. **Keiki Shimizu:** writing – review and editing. **Wataru Kasahara:** writing – review and editing. **Jun Hamaguchi:** writing – review and editing.

## Funding

The authors have nothing to report.

## Consent

Written informed consent for the publication of the details of this case was obtained from the patient's family.

## Data Availability

Data sharing not applicable to this article as no datasets were generated or analysed during the current study.
